# Application of protection motivation theory to clinical trial enrolment for pediatric chronic conditions

**DOI:** 10.1186/s12887-020-2014-5

**Published:** 2020-03-16

**Authors:** Stephanie P. Brooks, Tania Bubela

**Affiliations:** 1 Department of Pediatrics, University of Alberta, 3-62A Heritage Medical Research Centre (HMRC), 11207 - 87 Ave NW, Edmonton, AB T6G 2S2 Canada; 2 Faculty of Health Sciences, Simon Fraser University, Blusson Hall 11328, 8888 University Drive, Burnaby, BC V5A 1S6 Canada

**Keywords:** Pediatric clinical trial, Diabetes, Inherited retinal disease, Stem cell therapy, Gene therapy, Risk communication, Informed consent, Protection motivation theory

## Abstract

**Background:**

Parents of children living with chronic but manageable conditions hope for improved therapies or cures, including Advanced Therapy Medicinal Products (ATMPs). Multiple pediatric clinical trials for ATMPs are underway, but the risk profile of ATMPs for chronic conditions is largely unknown and likely different than for terminal pediatric illnesses. Applying Protection Motivation Theory modified to the context of pediatric ATMP clinical trial enrollment, our study analyses information needs of parents of children living with chronic manageable conditions: Type 1 Diabetes (T1D) or Inherited Retinal Diseases (IRD).

**Methods:**

We conducted semi-structured interviews with 15 parents of children living with T1D and 14 parents of children living with an IRD about: a) family background and the diagnostic experience; b) awareness of gene and stem cell therapy research and clinical trials for T1D and IRD; c) information sources on trials and responses to that information; d) attitudes to trial participation, including internationally; e) understanding of trial purpose and process; and f) any experiences with trial participation. We then discussed a pediatric ATMP clinical trial information sheet, which we developed with experts. We applied directed qualitative content analysis, based on PMT, to examine the information preferences of parents in deciding whether to enrol their children in stem cell or gene therapy clinical trials.

**Results:**

Parents balanced trial risks against their child’s ability to cope with the chronic condition. The better the child’s ability to cope with vision impairment or insulin management, the less likely parents were to assume trial risks. Conversely, if the child struggled with his/her vision loss, parents were more likely to be interested in trial participation, but only if the risks were low and likelihood for potential benefit was high.

**Conclusions:**

Fear of adverse events as part of threat appraisal was the predominant consideration for parents in considering whether to enroll their child living with a manageable, chronic condition in a pediatric clinical trial of an ATMP. This consideration outweighed potential benefits and severity of their child’s condition. Parents called for available safety data and fulsome communication processes that would enable them to make informed decisions about clinical trial enrolment on behalf of their children.

## Background

Many parents of children living with chronic but manageable conditions follow research advances, hoping for improved therapies or cures [1–3]. Experimental therapies include stem cell and gene therapies (collectively, Advanced Therapy Medicinal Products (ATMPs)). Multiple pediatric clinical trials for ATMPs are underway for a range of chronic conditions, and some are receiving regulatory approvals. Spark Therapeutics, recently by acquired by Roche, received United States Food and Drug Administration approval for its Leber congenital amaurosis gene therapy, including pediatric use [4]. Despite their promise, the risk profile of ATMPs for chronic pediatric conditions is largely unknown. This risk profile is likely different than for terminal pediatric illnesses, such as hematopoietic stem cell transplantation for acute lymphocytic leukemia.

**Fig. 1:**
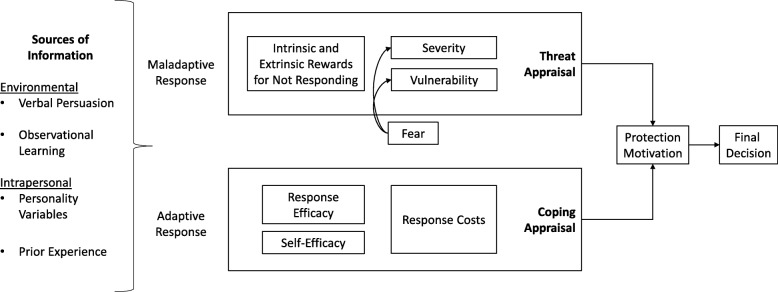
Protection Motivation Theory (adapted from Rogers 1983)

As the number of pediatric clinical trials for chronic pediatric conditions increases, parents question whether to enroll their children or to access unproven therapies at for-profit clinics around the world. It is timely, therefore, to assess the information needs of parents who are making health decisions on behalf of their children. We apply a well-established communication and behaviour prediction framework, the protection motivation theory (PMT) (Fig. 1) [5, 6], to analyse how parents use various forms of information to assess how to best protect an aspect of their child’s health or safety. Our qualitative, interview-based study focuses on parents of children living with chronic, manageable conditions: Type 1 Diabetes (T1D) and inherited retinal diseases (IRDs). T1D is managed by way of careful, constant monitoring and response to insulin levels with an insulin regimen delivered by injection or pump; IRDs are managed with environmental design of home and workspaces along with supportive aids for progressive visual impairment. Treatment regimens and/or accommodations for both T1D and IRDs are accordingly burdensome for children and caregivers.

Clinical trial outcomes for ATMPs receive significant media coverage, and media are primary sources of treatment information for patients, caregivers, and advocacy organizations [2, 7]. However, media coverage often underreports risk and overreports benefits and therapeutic potential of ATMPs [8–10]. Such coverage may generate unrealistic expectations for patients and families. For example, men living with one IRD, choroideremia, generally had overly optimistic views of the potential for gene therapy associated with overly positive media framing of clinical trial outcomes [8, 11]. Other sources feeding these views may include informed consent processes, as potential trial participants or their guardians misestimate the potential for therapeutic benefit [12], especially in early, safety-focused Phase 1 trials. Trial participants often conflate research and treatment and are frustrated by uncertainties over whether treatments will be available and affordable in a timely manner, if at all [13, 14].

Slow progress on pediatric ATMPs has led parents to seek alternatives. For example, frustrated by the slow approval of the Medtronics artificial pancreas system [15], waiting parents of children with T1D developed a community of ‘Health Hackers’ [16]. Under the hashtags #WeAreNotWaiting and #DIYPS (do-it-yourself pancreas system), families created closed loop, automated insulin delivery systems by sharing open access code combined with readily available health tracking technologies, such as mobile trackers and smartphones, to automate their insulin delivery based on basal rate tracking [17]. Open access code is made available by developers under terms that allow it to be modified and broadly shared. Such community modification of automated insulin delivery systems by-passes the regulatory environment for safety and efficacy of medical devices. Similarly, families seek unproven stem cell “therapies” for chronic diseases, including IRDs, from unregulated, for-profit clinics around the world, which market directly to consumers (a trend known as stem cell tourism) [18–20]. Such stem cell interventions have documented risks, including tumor development, disability, and death. Three women were recently blinded after receiving an unproven stem cell treatment, outside of a clinical trial, for age-related macular degeneration at a Florida clinic at a cost of 5000 USD per procedure [21]. Media coverage of family fundraising indicates that the cost for a child to receive a stem cell treatment at such a clinic for a chronic condition, including blindness, is in excess of 40,000 USD [18, 19].

As a result, researchers are advocating for guidelines to assist clinicians and clinical investigators in effectively communicating with patients about the risks and benefits of experimental or unproven ATMPs [13, 22, 23]. To date, studies of clinical trial communications have focused on fatal and acute pediatric conditions, for example in oncology and emergency medicine on how to deliver informed consent content [24–27], design informed consent forms [24, 28–30], and develop assent procedures [31]. Our study augments this literature by focusing on communications about enrolment in pediatric clinical trials of ATMPs for chronic manageable diseases.

### Theoretical framework

We relate our findings to the established risk communication theory, protection motivation theory (PMT) [32]. PMT helps explain information use in protective health decision-making processes. In this study, we focus on how parents use information on clinical trials that might slow or reverse negative health outcomes for their children who are living with a chronic, manageable condition. The PMT proposes that health protection motivation depends on a threat appraisal and an assessment of available options to cope with the threat.

To date, PMT has been used to understand how to encourage individuals to take action to avoid contracting or developing a disease (e.g. vaccination [33], exercise [34], smoking cessation [35]. In these conventional contexts, the threat appraisal is based on three factors: (a) perceived severity of the consequences of contracting or developing a disease; (b) perceived vulnerability, defined as the believed likelihood of contracting the disease; and (c) maladaptive response rewards, defined as the inherent rewards of not performing the recommended health protection method. Similar to the threat appraisal, the coping appraisal process uses three factors to assess recommended health protection behaviors: (a) response efficacy, defined as the degree of belief that the recommended responses will ensure avoidance of the threat; (b) self-efficacy, defined as the confidence in one’s ability to perform the recommended behavior; and (c) response costs and barriers, defined as the perceived financial, time, or energy-based costs required to perform the recommended health protection behavior [32]. Health protection decisions are finalized after assessing the severity of the threat against the ability to cope with the threat using the recommended methods. Thus employing the PMT enables understanding of how individuals weigh the costs and benefits of different health protection options to come to final behavioural decisions.

Parental decision-making for enrollment in pediatric clinical trials for chronic manageable diseases offers a complex example for the application of PMT. We describe how our study results informed modifications to the PMT. The modifications accounted for complexities in parental decisions about enrolling their children, who are living with chronic manageable diseases, in clinical trials for gene therapy and stem cell interventions.

## Methods

### Participants

Between October 2015 and January 2017, with the assistance of Canadian patient advocacy and research organisations (Foundation Fighting Blindness; Canadian National Institute for the Blind; Canadian Deaf Blind Association; Juvenile Diabetes Research Foundation; Canadian Diabetes Association), we recruited 15 parents of children under 18 years old living with T1D (*n* = 16) and 14 parents of children living with an IRD (IRD) (*n* = 16) across five English-speaking Canadian provinces (see Table 1). Children living with IRDs represented a spectrum of conditions and diseases including Usher’s syndrome (*n* = 2), retinitis pigmentosa (*n* = 3), Stargardt’s macular degeneration (3), Leber’s hereditary optic neuropathy (*n* = 1), retinoschisis (*n* = 2), Leber congenital amaurosis (*n* = 3), and achromatopsia (*n* = 2). The organizations enabled us to recruit at patient-focused conferences/research days by providing us with information booths to connect with potential participants directly or by providing study information letters in registration packages at conferences that we could not attend personally. Organizations also informed potential participants of the study via their online communication channels (e.g. newsletters). Participants who contacted the study staff provided informed consent prior to being interviewed.

**Table 1:** Characteristics of parents of children living with Type 1 Diabetes (T1D) or an inherited retinal disease (IRD) and characteristics of their children living with T1D and IRD

Parent Characteristics	Number of Parents		
	T1D	IRD	Total
**Number of children in parent family living with T1D or an IRD**			
1	14	12	26
2	1	2	3
3+	0	0	0
**Total**	**15**	**14**	**29**
**Number of years parenting a child living with T1D or an IRD**			
< 1	2	1	3
1–4	8	7	15
5–7	1	1	2
8–10	2	2	4
11–13	1	3	4
14–17	1	0	1
**Total**	**15**	**14**	**29**
**Prior family history of T1D or an IRD**			
Yes	4	3	7
No	11	11	22
**Total**	**15**	**14**	**29**
**Province of residence**			
Alberta	6	5	11
British Columbia	0	1	1
Northwest Territories	0	1	1
Ontario	9	6	15
Saskatchewan	0	1	1
**Total**	**15**	**14**	**29**
Child Characteristics	Number of Children		
	T1D	IRD	Total
**Children’s age at time of interview (years)**			
Under 5	3	1	4
5–10	4	6	10
11–17	9	9	18
**Total**	**16**	**16**	**32**
**Children’s age at diagnosis (years)**			
Under 5	9	10	19
5–10	5	5	10
11–17	2	1	3
**Total**	**16**	**16**	**32**

### Data collection

We conducted semi-structured interviews either in-person or over the phone, ranging from 15 to 70 min in duration (see Additional file 1). Our interview guide first addressed: a) family background and the diagnostic experience; b) awareness of gene and stem cell therapy research and clinical trials for T1D and IRD; c) information sources on trials and responses to that information; d) attitudes to trial participation, including internationally; e) understanding of trial purpose and process; and f) any experiences with trial participation. Our interview guide then asked parents to imagine that a researcher had approached them to enrol their child in a gene or stem cell therapy clinical trial. Our goal was not to test knowledge but to understand the substance and form of communications preferred by the parents. The questions allowed us to probe participants’ experiences with diagnosis and disease management and how communication of experimental or unproven treatments could be improved.

Finally, we presented the parents with either an information sheet for either a pediatric clinical trial of a stem cell therapy for T1D or a gene therapy for IRD, as appropriate (see Additional file 2). To ensure accuracy, we collaborated with clinical investigators on trials that were underway or planned in adults at the University of Alberta: *An Open Label Clinical Trial of Retinal Gene Therapy for Choroideremia* (NCT02077361) and *Stem Cell Mobilization (Plerixafor) and Immunologic Reset in Type 1 Diabetes* (T1DM) (NCT03182426). The information sheets addressed the trial background and purpose; and described the procedure; possible benefits; possible risks, including surgical risks; confidentiality; voluntariness of participation; and costs of and compensation for participation. The interviewer reviewed the information sheet with the parent and recorded all questions, concerns, reactions, and terms or concepts that required clarification.

### Data analysis

We used NVivo 11 qualitative analytic software to organize, manage, and analyze verbatim transcripts of the audio recorded interviews. We applied directed qualitative content analysis, based on the PMT, to examine the information preferences of parents in deciding whether to enrol their children in stem cell or gene therapy clinical trials. Directed content analysis is a common approach to healthcare research [36] in which transcripts are coded both deductively and inductively to test if/how data fits into existing theories or models [37, 38]. We started the study deductively coding transcripts using coding categories derived from the PMT. Deductive coding allowed us to examine similarities and differences between our participants and existing literature. We augmented our deductive coding with inductive coding, using a constant comparison approach [39] by immediately coding each interview transcript, adding new codes to the coding frame, and using the modified coding frame to review subsequent interviews, and re-analysing the already coded transcripts for the new codes.

To assess reliability of our analysis, a second investigator reviewed the codes to ensure that they comprehensively captured the key themes. We also conducted a member checking exercise [40]. We constructed a summary report for each interview and returned this to the parent to review the summary and return any comments, add or subtract material or ask additional questions. We integrated comments into the final analysis.

## Results

### Modified protection motivation theory (PMT)

**Fig. 2:**
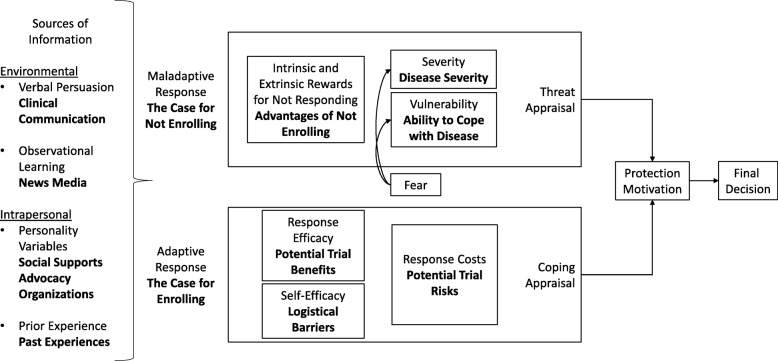
Protection Motivation Theory Constructs Aligned with Clinical Trial Contexts

Using the directed content analysis method, we modified PMT to account for the context of ATMP clinical trials for chronic, manageable, pediatric conditions (Fig. 2). Our deductive coding enabled us to identify interview data aligned with existing PMT constructs. Our inductive coding then allowed us to modify PMT to better represent the constructs in a clinical trial context (e.g. ‘maladaptive response’ became ‘the case for not enrolling’) (Fig. 2).

**Fig. 3:**
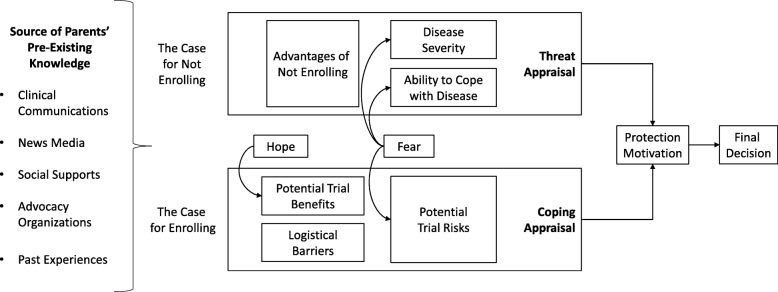
Modified Protection Motivation Theory in the Context of a Clinical Trial

We added two additional constructs to explain nuanced dynamics in parental decision-making. These were hope and fear and their effects in the adaptive response. In a clinical trial context, the threat appraisal makes the case for not enrolling in a clinical trial. The child continues to live with T1D or an IRD and does not assume the risks associated with clinical trial participation. The coping appraisal makes the case for enrolling in a clinical trial, based on potential benefits weighed against potential risks and logistical barriers. Parents simultaneously conducted a threat appraisal and a coping appraisal when considering the potential risks and benefits of clinical trial participation for their children (Fig. 3).

The interviewed parents used available information to evaluate the benefits and risks of clinical trial enrolment for their child, and which decision was in the best interests of their child. Below, we provide illustrative quotes for each construct of the modified PMT, starting from ‘Sources of Parents’ Pre-Existing Knowledge’ and ending with their ‘Final Decision’.

### Sources of parents’ pre-existing knowledge

Parents in our study were familiar with research on relevant gene and stem cell therapy research. Parents of children with T1D followed research on, islet encapsulation (*n* = 9), the artificial pancreas project (*n* = 7), and islet transplantation (*n* = 2). Parents of children living with an IRD reported following research on stem cell transplants (*n* = 7), gene therapies (*n* = 7), drugs (*n* = 4), CRISPR Cas9 (*n* = 2), bionic eye devices (*n* = 1), and homeopathic techniques (*n* = 1). Some parents were very active information gathers following a variety of information sources including news print, television and online news media and health providers. They often followed research foundation newsletters, such as those from Juvenile Diabetes Research Foundation or Foundation Fighting Blindness. Some also participated in online parent groups on Facebook or other social media platforms.

I follow a bunch of different kinds of organizations, groups and companies, the likes of Facebook, and the news and things. We read anything that pops up on things like the T1D page or [Juvenile Diabetes Research Foundation] page. We’re reading all the news releases and articles to see what’s in the pipeline, what people are looking into. – T1D

All had followed research news on their child’s condition, but some reported that they currently do not follow any research news (*n* = 3).

I searched like crazy after [my child’s] diagnosis. But honestly, I haven’t lately. I hear about things being planned but I kind of feel like it’s far away and it’s not something that’s realistic for the population right now. I’m just kind of waiting for to hear, I guess mainstream news. – T1D

Finally, seven parents said they had past experiences that informed their understandings of T1D or IRDs by way of family members or friends (Table 1). While other parents were surprised by their children’s diagnoses, those with past experiences had optimistic views of disease management derived from evidence of coping by family members.

Because of the family history, we were comfortable because his – a couple of his uncles actually do have it … we talked to [his uncle] and he has had a very successful business in Saskatoon and he does well in life. He has had 4 children … you do have an impairment but you’re still functional, right? – IRD

[The diagnosis] was tough but it was not end of the world because my sister at [her diagnosis] was 20 and obviously had led a normal life. She had some episodes where she ended up in the hospital but she has no complications from it as of yet. So I think that that helps … I remember when my sister was diagnosed my folks were devastated because we did not know anything about [diabetes] and nobody else had had it... whereas, at least I had a good support network and it was already in the family. – T1D

However, the parents did not have specific knowledge of our hypothetical trials of ATMPs for T1D and IRDs, respectively.

#### Clinical communications

Prior to reading the clinical trial information sheets, most parents did not understand the clinical trial process, nor did they have a clear understanding of preclinical and clinical research on the ATMPs. They required explanation before being able to discuss the evidence in support of clinical trials. While reading through the hypothetical ATMP clinical trial information sheets, parents asked for clarification on procedures, risks, and benefits. After reading the sheets and asking for clarification, parents were able to clearly explain these topics to the interviewer. Notably parents understood the difference between proven and unproven, between experimental and treatment, and could distinguish between hope and expectation.

I would want to know the purpose, are they giving them a really low dose? Is it just sort of a safety dose? Should we now expect to see any improvement or are they giving them the dose that they determine to be the most effective and be best results? – IRD

I understood that it was kind of experimental or a trial … It wasn’t tried, tested, and true. Because we use the words “trial” or “experiment”, I wasn’t optimistic about it... I went into it without expectation. – IRD

While parents generally started the interview optimistic about the potential for ATMPS, they often became less enthusiastic when they learned about the risks of trial participation.

So it’s a little bit scarier when you read [the information sheet] for sure. I mean I am more hesitant now than I was 20 min ago. – T1D

Okay. Oh my god. I do not know. There is one part that sounds yes exciting, like they could do it. But when I start reading the risks and all these things and everything, I do not like that part at all. – IRD

I do not know [whether I could say yes or no]. After reading the sheet it fully kind of changes my mind, make me hold on for a sec … I could not enter right now. I would have to really think about it and definitely talk to more professionals and really give it a lot of thought. – T1D

### The case for not enrolling: the threat appraisal

The case for not enrolling encompasses the elements of the decision-making process that may result in the child continuing to live with a chronic disease while not assuming unjustifiable trial risks.

#### Advantages of not enrolling

Parents considered the advantages of not enrolling in the clinical trial, including not assuming trial risks or waiting for ATMPs with a greater safety profile, even if waiting resulted in continued deterioration of vision or vigilant insulin testing and management. Almost all parents were uncomfortable with enrolling their child in a first-in-human clinical trial but might have considered a later stage trial with a pediatric safety and or efficacy profile.

We have always said that we don’t want our child to participate in early stage trials. We want it to be pretty much a sure thing before we subject our child to something. – IRD

I definitely would not want him to be one of the first people … If it had been tested on other, maybe not even people, I hate to say, “Oh, someone else’s kid has to test it out before mine” because that is obviously selfish because somebody has got to step up, but I would have to assess the risks. – IRD

#### Severity of and vulnerability to T1D/IRD

Parents used available information to assess the severity of the T1D/IRD against their child’s vulnerability or probability of experiencing severe consequences that have consequences for their wellbeing. The child’s ability to cope with the disease is what dictates whether assuming trials risks are justified.

I would rather maintain [my son’s] current eye sight rather than take a risk of losing all of it. He knows how to live with what he has now, so if we could maintain that forever I think we would be OK. He hates it, but he knows how to survive. – IRD

I think we’re managing a condition that’s is manageable and the risks are real, they’re known and largely controllable. And I think you would be trading that for known but uncontrollable risk. *So that doesn’t seem like a fair trade off.* – T1D

Similarly with established PMT constructs, fear informed both how parents considered disease severity and the ability to cope with the disease. The progressive nature of many IRDs caused fear among parents because there is a therapeutic window within which to try experimental treatments that may slow, halt or reverse vision loss. The threat of diabetic coma or long-term impacts of diabetes caused distress among parents.

“We check their glucose levels every night at midnight then again at 3 am … They’re on pumps now but we had a scenario 6 years ago … in theory we should not have had to check them … but I woke up at 4 am and checked and they were both at [dangerous lows]. And so there was no rhyme or reason … and so we decided at that point, it didn’t matter what equipment they were on, we would be checking them.” – T1D

### The case for enrolling: the coping appraisal

#### Potential trial benefits

Parents made clear that they were only interested in trials that were likely to provide direct therapeutic benefits. Parents were not interested in trials designed with placebo arms, because of wished for benefit in exchange for assumption of risk.

I would not want to get heavily involved in a trial where my kids might be on the placebo end of things, and I know that sounds ridiculous … There’s a really limited timeframe, you know, for the next few years and knowing the amount of time that it takes for research projects to progress, they don’t have time to waste**,** being the control arm. – IRD

In ophthalmic clinical trials, the design may involve a treated eye and a control eye in the same participant [41]. Parents acknowledged that this trial design reduced risks, but also perceived that the potential benefit was halved if the control eye was left untreated.

Well, I would assume that if it was safe after a certain amount of time that they would say “Hey, you’re up for the other eye now.” That is what I would assume would happen. I would not assume that they would just leave you if it worked and after a certain amount of time. – IRD

I like that. I like that. Assuming that you could get the other eye treated after. – IRD

Similarly, parents were concerned about reversible treatment for T1D (e.g., devices or implants that would be tested and removed at the end of the trial). Some parents appreciated that if beneficial, the implant could potentially give their child a period of normalcy during their childhood. Others were uncomfortable with reversing the treatment if it proved beneficial.

They stop the treatment after 3 years regardless of how well it’s working? My heart dropped there … once you see that benefit, you want to keep seeing benefit. – T1D

#### Hope

Hope informed the appraisal of potential trial benefits but was viewed by parents as a double-edged sword. Parents found hope to generate good feelings and to be a helpful coping tool,

I am just thinking from the other view - you want to kind of temper people’s expectations, but from a parent’s point of view, we want hope. – IRD

I mean we have talked to [my son] about it too and he’s like, “Well, yeah I get it Mom but, you know what happens if it works?” [and I said to him] “But you know there is a lot of risks involved.” [and he replied] “Yeah, I know but that’s okay but what happens if it works?” – IRD

However, parents also recognized the pain of false hope and they did not want to inflict false hope on to their children by accident.

Interviewer: So, you have never talked about trials with your daughter?

Parent: No. Because I do not want to get her hopes up … I do not know if she fully understands her condition yet, but I do not want to plant that seed because I do not even know if this ever could happen. – IRD

[Learning about CRISPR] felt other worldly and I felt like it was too good to be true … I felt very, very hopeful, but I was also somewhat skeptical … and fearful because I did not want our hope to be raised up and our child hopes. We never mentioned it to [my son]. – IRD

It’s more about managing emotional risks than physical ones in some ways. I’m not willing to take a try-anything-and-everything-approach, because [my son] could be set up for heartbreak. – T1D

#### Logistical barriers

Logistical barriers may prevent clinical trial participation. Determination to overcome logistical barriers is influenced by perception of likelihood and magnitude of potential benefit. Only one parent stated that logistics would be a barrier to trial participation. All others stated the intention to do anything if the magnitude and likelihood of benefits were high enough.

Well, I could and would if I was committed to the process, like I am not committed to the process at the moment. So I cannot say ‘I will’ but I could, yes. And I would if that was what was – I was committed to do for my daughter, yeah. I would go to the end of the earth to help her. – IRD

If there was something out there and someone says, “Hey, you know what for a million dollars I can fix your son guaranteed 100% no side effects,” right? If I don’t have it, I’ll work my ass off until I bleed in order to get that surgery for my son. So that he can have the best life possible. That is when I would step up and do whatever to get there. But to put risk on him, that is a whole different ball game. – IRD

I would pay anything. I don’t care where it is or how much it is. I would have paid to go find it. Like if my son can be cured of T1D, I would pay for it today. I don’t care how much it is. I can find it. – T1D

#### Trial risks – physical

Parents were realistic about the potential risks of ATMP clinical trials, including loss of vision, need for immunosuppressant, disqualification from future research or treatment, child suffering, tumor development, lack of long-term data, high drug use requirements, and diabetic ketoacidosis. While fear is not normally associated with PMT’s ‘response costs’ construct, it is present in trial contexts as the experimental intervention is inherently risky. Given the risk and associated fear, parents wished to make the best decisions on behalf of their child and avoid potential guilt for making a poor decision.

… but permanent loss of eyesight, as soon as they are telling me this, that’s probably the big turn off that I would say no. Like no. Probably I would never forgive myself if she went into surgery and then she lost whatever she has left. I would never forgive myself ever. – IRD

… if there is a side effect that could cause problems, I could never put my child through that. I could never be that guy and be like oh I was that parent that said, “Yeah” and now, now this has happened. I could never live with myself. – IRD

I don’t really want to take any risks, anything that’s going to hurt him any further because God forbid with some other things that I should do something that makes him lose more of his vision. – IRD

How do you - If you made matters worse, you know, how do you explain that to your child and live with yourself and so on? So that was the kind of thinking that was going on in my head. – T1D

#### Trial risks - opportunity costs

Almost every parent commented that trial participation may exclude their child from being eligible for future trials or approved treatments.

One of the things is that if she participated in this study then that might preclude her from doing other studies. So I guess I would want to know what other studies are being done and what you foresee as how the study rates against the others for success. Because then I might want to wait for another study as opposed to participating in this one. – T1D

### Protection motivation

Protection motivation is the outcome of assessing the threat and coping appraisals. These appraisals enable parents to decide whether they are more motivated to protect their children from disease severity or from trial risks. In other words, parents balanced trial risks against their child’s ability to cope with the chronic condition. The better the child’s ability to cope with vision impairment or insulin management, the less likely parents were to assume trial risks. Conversely, if the child struggled with his/her vision loss, parents were more likely to be interested in trial participation, but only if the risks were low and likelihood for potential benefit was high.

### Final decision and information required for informed consent

Parents called for complete information about risks, benefits and uncertainty to help them make decisions about safety and efficacy trials of experimental ATMPs.

I was really excited about it at first. I still am. I’m still very excited about it. Of course, there’s always the worries. Will it make it worse? There’s always that. What are the repercussions of it? What will happen if he was one of those people that doesn’t respond to it correctly or that kind of thing? – IRD

Most parents expressed a preference to talk to the lead clinical researcher before making an enrollment decision, but there was no expectation that the clinical lead be the first person to contact the families nor the person to lead the informed consent process. First, parents wanted to speak directly to the research lead to clarify the risks and uncertainties for their child.

I have always appreciated that [my daughter] was present when information was given … then I don’t have to re-explain it or give my twist on it … I’ve always been thankful that she has been present so that she hears what I hear and then we make decisions. – IRD

I would want to talk to the actual doctor that’s been doing this to find out, because I’m not a medical professional, I would want to know what type of risks could be possible in children. I know that they have not studied it in children and they can’t say for sure … but I would need to hear the ways that the body grows and what having a device in there may or may not do. – T1D

I think an assistant is the first. I think that is fine. I think that is a fine first contact but ultimately, I want to talk to the lead researcher. – IRD

Second, parents felt that their child needed to trust the clinical research lead and feel comfortable with the research team before the parents would consent to the research.

I would want the option to bring him in and have him – again have the opportunity to see where the procedure would take place and meet the doctors and to be comfortable. – T1D

Parents’ request for their children to meet the clinical research lead was related to their desire to include their children in the decision-making process. Most parents wished to include their child in the final decision or to have the child make the decision.

I would have to talk to him really seriously. My yes is dependent on his yes. – T1D

[My son], he does have a lot to say. He would have a lot of influence on any decisions … I would want the person running the trial, because I’m a parent who makes sure that [my son] is always in the room so he can ask as many questions as he could. Because it is his body. – IRD

I think that those conversations would happen at every age at the point they can understand … I don’t know. But at 4, he certainly can be involved in decisions, like “this is going to happen, how do you feel about it? Do you feel strongly about it?” Okay, if so we’re probably not going to proceed. – T1D

Many parents expressed the need for a second opinion before making any final decisions, in some cases from spouses or trusted family members, in others from their child’s health care provider. Many also wished to speak with prior trial participants to gain a more holistic understanding of the experiences they could expect for their children.

Talking to the actual patient you could say, “Well, yeah that it hurts but it was worth the hurt because I was able to do this.” Where I do not think doctors are able to really say that unless they are actually going through it right? – T1D

100% I would want to talk to the doctors performing the research. Then anyone else going through it, in the trial … some who’s actually living the experience … some type of opportunity to listen face-to-face, because it is one thing to read it and another to engage in the discussion. – T1D

Absolutely, [my son], I would want him involved as well as my husband. And maybe my dad. He’s a big science guy so I think he would maybe hear something different than me as a parent. Where he would be a little more objective more maybe more cautious. – IRD

### Treatment tourism

Most of the parents would not enable their child to receive unapproved therapies outside of the context of a credible clinical trial, including in a country with lower regulatory standards than North America or Europe.

No. I mean, yeah, obviously, the credibility of the researchers— It kind of goes along with the chances of this being actually successful. You have to trust the researchers. You don’t have time to waste on something that’s not credible. – IRD

No, if we are talking about China, no. Because I just find that their ethics are not up to my standard and I think they just do things too quickly and yeah, no. I would stick to Canada. – IRD

You do not go online and type in ‘diabetes research’ because you get all kinds of crap and I am not going to Mexico for some weird clinic, some alternative therapy. I’m just not … It’s too Cracker Jack for me. If [my son] was going to participate in a trial, there would have to be some evidence that supported success. – T1D

However, one parent had taken her son overseas for an unproven homeopathic treatment, after engaging in the same decision-making process that involved both threat and coping appraisals as described above. The parent of a child with an IRD felt hopeless because there was no treatment available in North America. Online, she found a clinic that represented it could cure her son’s condition through patient testimonials.

When we looked on the website, they said it’s permanent, there was no risk they have been doing it for so long and then like I said, I talked to patients. I found them on the website or on Facebook and I contacted them and that is when we talked to patients … They had good results, that is when we, we felt comfortable, like nerve wracking but no pain and that stuff for the child. That is when we felt like, my son is not the only one, there’s a lot like him. [The clinic] has been doing it from 2001 and there is nothing but amazing comments. – IRD

Her son’s clinician in Canada did not explain any risks that might have tempered the positive framing on the clinic website and conversations with past patients.

I did talk to [my clinician’s] specialist, and she said “Well, I cannot say yes and I cannot say no. That’s your decision. We don’t know about [the clinic].” And there is pretty much no cure, so who else am I going to talk to? – IRD

In her view, she felt her son’s vision improved after the treatment, and she submitted this result to the provincial government. She was frustrated that Canada would not approve the treatment and was confused and frustrated that the Province found there was insufficient evident to pay for the treatment.

However, when presented with information about the risks and benefits of ATMP clinical trial enrolment, she demonstrated the same hesitancy as other parents.

For me honestly, I would be scared to put my child into this clinical trial … because it is saying it is an operation, right? That will inject fluid under the retina. I do not know, if it was to be after the treatment in the clinical trial was approved then I would. – IRD

## Discussion

Using the modified PMT, our results, when compared to those of other studies, demonstrated distinctions in decision-making processes between: a) preventative and treatment behaviors; b) acute/fatal diseases and chronic manageable conditions; and c) personal decisions versus those made on behalf of someone else. Protective behaviours studied in health contexts are most commonly those to detect diseases (e.g. breast cancer screening), to adopt health practices (e.g. following an exercise regimen), or to cease unhealthy behaviours in response to acute or reversible disease threats (e.g. smoking cessation) [42, 43]. Clinical trial enrollment decisions are different in a number of fundamental ways.

Seeking treatment in general is an understudied behavior in PMT research [43]. To our knowledge, this is the first study applying PMT for those seeking experimental interventions. Furthermore, PMT literature often focuses on decisions to take up safe and healthy alternatives to poor existing health behaviours. Participants in our study viewed clinical trial enrollment is a potential alternative to living with T1D or and IRD but recognized the risks involved in experimental interventions.

Normally, response costs (trial risks in our PMT modification) represent beliefs about how costly performing the new protective behavior will be [42] (e.g. energy required to perform an action, comfort disclosing health information), however they are not normally risky themselves. Normally PMT is applied to situations where a person can continue with an unhealthy behavior or adopt a healthier path. Clinical trial contexts present a case for decisions between two risky paths containing uncertain outcomes. This is an exceptionally difficult type of decision to make on someone else behalf, especially when physical safety is at risk [44], as was the case for the parents in this study.

Parents relied on existing values and knowledge in making health-related decisions on behalf of their children. They desired complete information on risks and potential benefits before making any clinical trial enrollment decisions. Parents’ approach to decision-making reflected the nature of their children’s manageable, chronic conditions. Similar to other studies, our parent participants were prepared to carefully consider acceptable risks weighed against benefits [45, 46]. Parents in our study frequently articulated that their children were coping with their conditions, making decisions to enroll in clinical trials harder to justify.

### Application of PMT to pediatric clinical trials for chronic manageable conditions

The threat appraisal informed the case against enrolling in a trial. Considerations included protecting a child from further discomfort or harm and waiting for more evidence of safety and efficacy before making a decision on behalf of a child. This intention to wait for better evidence contrasts with prospective adult clinical trial participants, who have little recognition and appreciation of risks and disadvantages of trial participation [47], suffer from therapeutic misconception [14], and overestimate potential benefits [48] when signing consent forms. However, parents are often anxious about their child being treated as a ‘guinea pig’ [26] and fear making the ‘wrong’ decision [49].

Under the PMT model, vulnerability relates to the probability of contracting a disease or condition if a behaviour is left unchanged. In our study, vulnerability related to the impact of the condition on the child’s quality of life. While parents hoped for improved therapies, their primary interest was to protect their children from further negative health and life impacts. Parents of children living with T1D sought to protect their children’s experience of childhood with treatments that improved diet, sleep, and need for insulin injections. Parents of children living with IRDs sought to protect the remaining vision of their children.

With respect to the coping appraisal, parents were receptive to considering the potential benefits of trial participation for their children, with primacy given to therapeutic benefit not altruism. Indeed, the hope for therapeutic benefit led one mother to engage in treatment tourism for her child [19, 20]. The ability to overcome logistical barriers is understood as a key component in all major health behavior models, including PMT [50], but our results suggest that, in a trial context, strong beliefs that the trial will create benefit negate logistical considerations. Other benefits not identified by our participants include free medication, better care of their child, and greater access to healthcare professionals and health information [45].

### Implications for pediatric clinical trial consent process

Contrary to concerns that parents may suffer from therapeutic misconception or unrealistic optimism [51, 52], our participants differentiated between hope and expectation. To facilitate this differentiation, pediatric clinical trial consent processes must provide complete information, including about: a) the scientific process and understanding uncertainty; b) maximum therapeutic potential; c) magnitude and likelihood of all benefits and risks; and d) approved and available supportive or treatment regimens. The need for transparency and completeness of information are compounded by trust relationships – mothers of children involved in diabetes research assumed benefits as they could not “imagine honorable nurses or physicians putting their children at risk” [53].

Our findings support the need for guidelines on communications about enrolment in legitimate pediatric clinical trials [13, 22, 23]. Such resources are available from research and patient organizations (e.g., Foundation Fighting Blindness [54] and International Society for Stem Cell Research [55]) and can help health care providers discuss clinical trial processes, expectations for therapeutic developments, realistic timelines, and how to identify legitimate clinical trials compared to unproven treatment tourism options. Communications should modulate expectations, while maintaining hope [13].

Similar to other studies, our parent participants additionally expressed the wish to contact prior participants to help inform their decisions [30, 45]. Such a practice could be permissible with consent of the participants to be contacted; however, it comes with a risk of misinformation and selection bias, which may distort the norm towards positive or negative experiences. Parents additionally suggested that they would like to discuss clinical trial participation with the clinical investigative lead, rather than with their physicians or other trial staff [42]. This is contrary to best practice, which insulates clinical investigators from consent processes in case of undue pressure on potential participants ([56], Article 3.1 (Undue Influence)).

### Study limitations

Our study included only participants from English-speaking Canadian provinces, with overrepresentation from Alberta and Ontario. Our recruitment biased our population towards those connected in-person or online to patient advocacy organizations. Our sample size did not enable us to distinguish parents’ responses based on the current age, age of diagnoses, time since diagnosis, and severity of the condition of their child. We conducted individual interviews with parents not with family units, including the children. Decisions to participate in clinical trials are unlikely to be made by individual parents. Further, we recruited participants who had a relationship with patient advocacy organizations; thus participants likely had an interest in clinical research and their views may not be representative of a general population of parents.

We reviewed clinical trial information with parents in a hypothetical context, which may not have elicited a full range of reflections. Our study did not follow up with parents and therefore could not assess duration of understanding on the risks, benefits, uncertainty, and process they articulated during the interview. Other studies recommend either an ongoing informed consent process or multiple meetings that revisit the risks and benefits prior to final consent for participation [57, 58].

## Conclusion

Novel ATMPs lack a strong evidence base of benefit and a well-characterized risk profile. However, expectation for benefit is high and public information sources often overstate benefits relative to risks. This places an onus on clinical investigators to engage in best communications and consent practices, especially in the context of pediatric clinical trials. Our results suggest that fear of adverse events as part of threat appraisal was the predominant consideration for parents in considering whether to enroll their child living with a manageable, chronic condition in a pediatric clinical trial of an ATMP. This consideration outweighed potential benefits and severity of their child’s condition. Parents called for available safety data and fulsome communication processes that would enable them to make informed decisions about clinical trial enrolment on behalf of their children.

## Supplementary information

**Additional file 1.** Semi-Structured Interview Guide.

**Additional file 2.** Hypothetical Clinical Trial Information Sheets.

## Data Availability

Interview transcripts are protected under the *Health Information Act* of the Province of Alberta. De-identified interview transcripts are available from the authors on request.

## Abbreviations

ATMP: Advanced Therapy Medicinal Product
IRD: Inherited Retinal Disease
PMT: Protection Motivation Theory
T1D: Type 1 Diabetes

## References

[1] Strekalova YA (2016). Finding motivation: online information seeking following newborn screening for cystic fibrosis. Qual Health Res.

[2] Lu C, Wirrell E, Blackman M (2005). Where do families of children with epilepsy obtain their information?. J Child Neurol.

[3] Dillard JP, Tluczek A (2005). Information flow after a positive newborn screening for cystic fibrosis. J Pediatr.

[4] FDA. FDA approves novel gene therapy to treat patients with a rare form of inherited vision loss. December 19 2017. https://www.fda.gov/NewsEvents/Newsroom/PressAnnouncements/ucm589467.htm. Accessed 28 Mar 2019.

[5] Rogers RW (1975). A protection motivation theory of fear appeals and attitude Change1. J Psychol.

[6] Maddux JE, Rogers RW (1983). Protection motivation theory and self-efficacy: a revised theory of fear appeals and attitude change. J Exp Soc Psychol.

[7] Lipstein EA, Lovell DJ, Denson LA, Moser DW, Saeed SA, Dodds CM, Britto MT (2013). Parents’ information needs in tumor necrosis factor-α inhibitor treatment decisions. J Pediatr Gastroenterol Nutr.

[8] Benjaminy S, Bubela T (2014). Ocular gene transfer in the spotlight: implications of newspaper content for clinical communications. BMC Med Ethics..

[9] Sharpe K, Pietro ND, Illes J (2016). In the know and in the news: how science and the media communicate about stem cells, autism and cerebral palsy. Stem Cell Rev.

[10] Elliott R, Rödder S, Franzen M, Weingart P (2012). The Medialization of regenerative medicine: frames and metaphors in UK news stories. The sciences’ media connection –public communication and its repercussions.

[11] Benjaminy S, MacDonald I, Bubela T (2014). “Is a cure in my sight?” Multi-stakeholder perspectives on phase I choroideremia gene transfer clinical trials. Genet Med.

[12] Kimmelman J (2009). Gene transfer and the ethics of first-in-human research: lost in translation.

[13] Benjaminy S, Kowal SP, MacDonald IM, Bubela T (2015). Communicating the promise for ocular gene therapies: challenges and recommendations. Am J Ophthalmol.

[14] Pentz RD, White M, Harvey RD, Farmer ZL, Liu Y, Lewis C (2012). Therapeutic misconception, misestimation, and optimism in participants enrolled in phase 1 trials. Cancer.

[15] FDA (2016). FDA approves first automated insulin delivery device for type 1 diabetes.

[16] Omer T (2016). Empowered citizen “health hackers” who are not waiting. BMC Med.

[17] Lewis D (2016). Frequently Asked Questions.

[18] Wood C (2011). Family finds hope in stem cell therapy. Coast Reporter.

[19] Wood C (2012). Rylan heads to China soon. Coast Reporter.

[20] Zarzeczny A, Caulfield T (2010). Stem cell tourism and doctors’ duties to minors—a view from Canada. Am J Bioeth.

[21] The Guardian (2017). Three women with eye disease blinded by unproven stem cell treatment.

[22] de Melo-Martín I, Hellmers N, Henchcliffe C (2015). First-in-human cell transplant trials in Parkinson’s disease: the need for an improved informed consent process. Parkinsonism Relat Disord.

[23] Greenberg J, Smith DC, Burman RJ, Ballo R, Kidson SH (2015). Towards guidelines for informed consent for prospective stem cell research : informed consent. S Afr J Bioeth Law.

[24] Shaw MG, Morrell DS, Corbie-Smith GM, Goldsmith LA (2009). Perceptions of pediatric clinical research among African American and Caucasian parents. J Natl Med Assoc.

[25] Sureshkumar P, Caldwell P, Lowe A, Simpson JM, Williams G, Craig JC (2012). Parental consent to participation in a randomised trial in children: associated child, family, and physician factors. Clin Trials.

[26] Caldwell PHY, Murphy SB, Butow PN, Craig JC (2004). Clinical trials in children. Lancet.

[27] Chappuy H, Doz F, Blanche S, Gentet JC, Pons G, Tréluyer JM (2006). Parental consent in paediatric clinical research. Arch Dis Child.

[28] Hoberman A, Shaikh N, Bhatnagar S, Haralam MA, Kearney DH, Colborn DK (2013). Factors that influence parental decisions to participate in clinical research: consenters vs nonconsenters. JAMA Pediatr.

[29] Barfield RC, Church C (2005). Informed consent in pediatric clinical trials : current opinion in pediatrics. Curr Opin Pediatr.

[30] Lebensburger JD, Sidonio RF, DeBaun MR, Safford MM, Howard TH, Scarinci IC (2013). Exploring barriers and facilitators to clinical trial enrollment in the context of sickle cell anemia and hydroxyurea. Pediatr Blood Cancer.

[31] Mammel KA, Kaplan DW (1995). Research consent by adolescent minors and institutional review boards. J Adolesc Health.

[32] Rogers RW, Cacioppo JT, Perry RE (1983). Cognitive and physiological processes in fear appeals and attitude change: a revised theory of protection motivation. Social psychophysiology: a source book.

[33] McNeill A, Harris PR, Briggs P (2016). Twitter influence on UK vaccination and antiviral uptake during the 2009 H1N1 pandemic. Front Public Health.

[34] Milne S, Orbell S, Sheeran P (2002). Combining motivational and volitional interventions to promote exercise participation: protection motivation theory and implementation intentions. Br J Health Psychol.

[35] Curry S, Wagner EH, Grothaus LC (1990). Intrinsic and extrinsic motivation for smoking cessation. J Consult Clin Psychol.

[36] Elo S, Kynagäs H (2008). The qualitative content analysis process. J Adv Nurs.

[37] Assarroudi A, Nabavi FH, Armat MR, Ebadi A, Vaismoradi M (2018). Directed qualitative content analysis: the description and elaboration of its underpinning methods and data analysis process. J Res Nurs.

[38] Mayring P (2000). Qualitative content analysis. Forum: Qual Soc Res.

[39] Charmaz K, Smith JA (2006). Grounded theory. Constructing grounded theory: a practical guide through qualitative analysis.

[40] Lincoln YS, Guba EG, Pilotta JJ (1985). Naturalistic inquiry.

[41] Maguire AM, High KA, Auricchio A, Wright JF, Pierce EA, Testa F (2009). Age-dependent effects of RPE65 gene therapy for Leber's congenital amaurosis: a phase 1 dose-escalation trial. Lancet.

[42] MILNE SARAH, SHEERAN PASCHAL, ORBELL SHEINA (2000). Prediction and Intervention in Health-Related Behavior: A Meta-Analytic Review of Protection Motivation Theory. Journal of Applied Social Psychology.

[43] FLOYD DONNA L., PRENTICE-DUNN STEVEN, ROGERS RONALD W. (2000). A Meta-Analysis of Research on Protection Motivation Theory. Journal of Applied Social Psychology.

[44] Stone ER, YoonSun C, Bruine de Bruin W, Mandel DR (2013). I can take the risk, but your should be safe: self-other differences in situations involving physical safety. Judgm Decis Mak.

[45] Caldwell PHY, Butow PN, Craig JC (2003). Parents’ attitudes to randomised controlled trials involving children. J Pediatr.

[46] Zupancic JAF, Gillie P, Streiner DL, Watts JL, Schmidt B (1997). Determinants of parental authorization for involvement of newborn infants in clinical trials. Pediatr.

[47] Lidz CW, Appelbaum PS, Grisso T, Renaud M (2004). Therapeutic misconception and the appreciation of risks in clinical trials. Soc Sci Med.

[48] Jansen LA, Mahadevan D, Appelbaum PS, Klein WMP, Weinstein ND, Mori M (2016). Dispositional optimism and therapeutic expectations in early-phase oncology trials. Cancer.

[49] Shilling V, Young B (2009). How do parents experience being asked to enter a child in a randomised controlled trial?. BMC Med Ethics.

[50] Maddux JE, Kleiman EM, Wood A, Johnson J (2016). Self-Efficacy: A Foundational Concept for Positive Clinical Psychology. The Wiley handbook of positive clinical psychology.

[51] Woods S, Hagger LE, McCormack P (2014). Therapeutic misconception: Hope, trust and misconception in Paediatric research. Health Care Anal.

[52] Horng S, Grady C (2003). Misunderstanding in clinical research: distinguishing therapeutic misconception, therapeutic Misestimation, & therapeutic optimism. IRB.

[53] Pletsch PK, Stevens PE (2001). Inclusion of children in clinical research: lessons learned from mothers of diabetic children. Clin Nurs Res.

[54] Foundation Fighting Blindness. Clinical Trials. October 11 2016. https://ffb.ca/everything-you-need-to-know-about-clinical-trials/. Accessed 18 Apr 2019.

[55] International Society for Stem Cell Research. Stem Cell-Based Clinical Trials: Practical Advice for Physicians and Ethics/Institutional Review Boards. January 23 2018. http://www.isscr.org/docs/default-source/clinical-resources/isscr-stem-cell-based-clnical-trials-practical-advice_final_23jan2018.pdf?sfvrsn=2. Accessed 18 Apr 2019.

[56] Government of Canada (2018). Tri-Council Policy Statement: Ethical Conduct for Research Involving Humans – TCPS 2.

[57] Foglia MB, Salas HS, Diekema DS (2009). A quality improvement approach to improving informed consent practices in pediatric research. J Clin Ethics.

[58] DCCT Research Group (1989). Implementation of a multicomponent process to obtain informed consent in the diabetes control and complications trial. Control Clin Trials.

