# Supplementary material for: Application of protection motivation theory to clinical trial enrolment for pediatric chronic conditions
Source: BMC Pediatr. 2020 Mar 16;20:123. doi: 10.1186/s12887-020-2014-5 (PMC7075002; doi:10.1186/s12887-020-2014-5)
Supplement: Supplementary file 1 — Additional file 1. Semi-Structured Interview Guide. [file 12887_2020_2014_MOESM1_ESM.docx]

**Interview Guide**

Description of data: Below is the semi-structured interview guide we used to facilitate the interviews with parents.

**Part 1: Current Experiences with Information and Perspectives of Novel Clinical Trial Interventions**

General Background

1. **Tell me a little about yourself and your family.**
2. **Tell me about your child’s [insert disease] and how it affects your day-to-day life as a parent.**
3. **Think back to when you first learned about your child’s diagnosis. Can you tell me about that experience?**

PROBING QUESTIONS:

1. What do remember about the first treatment or management options and recommendations?
2. How has the management regimen for your child’s [insert disease] changed over time?

[Probe for reasons why they chose to change management regimen]

Awareness and perspectives of gene and stem cell technologies for type 1 diabetes and genetic ocular diseases

1. **Have you heard about gene or stem cell clinical trials for [insert disease]?**

PROBING QUESTIONS:

1. How did you hear about the clinical trial(s)? Please provide specific sources.
2. Describe the coverage or conversation.

[Probe for tone, spin, trust, accuracy of the source and information]

1. Please describe your feelings when you first heard about these trials.
2. Did you consider enrolling your child in a clinical trial outside of Canada? [Probe on where?]
3. Did the way these trials were covered impact how you felt about potentially putting your child into one of these trials?
4. If so, what considerations persuaded/dissuaded you from putting your child into a trial like this?

[Probe for emotions, events, perspectives that led to these considerations. Why are these things important to the participant?]

1. **Can you describe why researchers conduct clinical trials?**

PROBING QUESTIONS:

1. What happens during clinical trials? Do you know what the clinical trial process looks like?
2. Tell me about these clinical trials and how they might affect treatment for your child’s condition in the future
3. Have you ever considered putting yourself or your child into a clinical trial? Why/why not?

[Probe for specific reasons that differentiate children from adults from the participant’s perspective]

1. **Do you know anyone who has enrolled their child in a clinical trial, either in Canada or in another country?**

PROBING QUESTIONS:

1. Can you describe how that person spoke about their experience?
2. How did you feel hearing about this experience? What resonated with you hearing about their child being in a clinical trial?

**Part 2: Hypothetical Trial Participation**

1. **Imagine a researcher asks to you enrol your child into a gene therapy OR stem cell transplantation clinical trial. The following questions are not to test your knowledge. I want to understand what you would want to know and understand and how you would like that information communicated.**

PROBING QUESTIONS:

1. What would you want to know about clinical trials before considering enrolling your child in a clinical trial of an experimental treatment?

[Probe risks and benefits]

1. How would you like that information communicated and by whom?
2. How would you feel emotionally about choosing whether or not to give consent for your child to participate in a clinical trial?

[Probe for reasons such emotions would emerge]

1. How do you think your child would react to being in a trial both physically and emotionally? Why do you think your child would react this way?
2. Do you think that clinical trial participation would change your child’s quality of life? If so, how?

**Part 3 – Case Based Scenario of a Clinical Trial Decision**

1. **Please read this consent form for a hypothetical clinical trial carefully and ask me any questions or raise any concerns that you have. I want to stress that at present clinical trials in Canada are not proceeding in children, and are only starting or being planned in adults.**

PROBING QUESTIONS:

1. What are your first reactions to this information sheet?

[Probe for what caused excitement, worry, confusion, etc.]

1. Based on what you just read, before we discuss the form, what would be your initial decision of enrolling your child in this trial? OR Would you be able to make a decision to enroll you child in this trial or not without discussing it with the researcher first? Please describe how you came to that decision.
2. Now, was there anything in this form that you would want clarification about?
3. Describe the risks involved with this trial.
4. How do you feel about the risks of this trial?

[Probe for perspectives, past events, reasons that the risks are or are not important to the participant]

1. Describe the benefits of the trial.
2. [Probe for perspectives, past events, reasons that the risks are or are not important to the participant]
3. Do you think the risks or benefits of this trial are greater? How so?
4. After reading this information and chatting about the form with me, would you consider enrolling your child in the trial? Why/why not?

[Probe for factors that affect parental willingness to enroll the child in the clinical trial]

1. **Is there anything else you would like to add?**
