# Supplementary material for: Application of protection motivation theory to clinical trial enrolment for pediatric chronic conditions
Source: BMC Pediatr. 2020 Mar 16;20:123. doi: 10.1186/s12887-020-2014-5 (PMC7075002; doi:10.1186/s12887-020-2014-5)
Supplement: Supplementary file 2 — Additional file 2. Hypothetical Clinical Trial Information Sheets. [file 12887_2020_2014_MOESM2_ESM.docx]

**Hypothetical Clinical Trial Information Sheets (3)**

Description of data: Below are three hypothetical information sheets we presented to parents as appropriate. The information sheets present risks and benefits of trial participation for a pediatric clinical trial. Parents used these sheets to reflect on what risks and benefits would be tolerable for their child.

**Information Sheet #1: An open label pediatric clinical trial of retinal gene transfer**

**Background:**

Right now, there are no effective treatments available for genetic retinopathies. But, a new technique of gene therapy is being developed, which we think may help to slow or even stop the decline of vision due to your child’s disease. The new technique puts working copies of the mutated gene into the cells of the retina to help them function. This is done by injecting the working genes underneath the retina. A modified virus carries the gene into the cells. The virus has been modified so that it cannot cause infection.

Gene therapy has been tested in a small number of adult and child patients with other retinal diseases caused by gene changes. The modified virus we use is similar to that which was used in these other clinical trials, but, in those trials, the virus carried a different gene. Clinical research with a small number of participants has shown that inserting the working gene into the retina of patients with another genetic retinal disease, called Leber congenital amaurosis (LCA), can help to preserve or restore some vision. Results show that the therapy was more effective in younger patients. However, we do not know how gene therapy will affect research participants with other retinopathies.

**Purpose:**

The main purpose of this study is to make sure that gene therapy is safe for children. The secondary purpose is to develop good methods for testing if gene therapy is effective in slowing or stopping vision loss. The data we collect will then be used to inform further clinical trials. You have been asked to consent on behalf of your child because he/she has the genetic retinopathy in which we are interested, and there is currently no proven treatment for this disease. We expect to follow research participants for 2 years after the treatment. For this study, we intend to include 12 participants.

**Procedure:**

As part of this clinical trial, your child will have an operation that will inject a saline fluid under the retina of one eye. The saline fluid contains the modified virus carrying a healthy version of the mutated gene that causes your child’s disease. The modified virus can no longer cause infection and is designed to simply deliver the human genes into the cells of the retina. This procedure involves surgery using local anesthesia to the eye. The surgeon will remove some of the jelly-like material that exists at the back of the eye. Then the surgeon will inject the saline fluid (a tenth of a millimeter or about 2 drops) under the retina. This type of surgery normally lasts about an hour and the operation itself is a routine procedure for patients with conditions such as retinal detachment. The surgical procedure will be videotaped to provide a record of the operation and determine its safety. The gene transfer will be given in one eye and the other eye will be untreated and used as a comparison.

Your child will also have tests before and after the operation. The tests will be vision tests which would normally have been performed as part of your child’s routine eye care. If your family does not live in Alberta, as many tests as possible will be performed at your referring physician’s centre so that you do not have to travel as much. However, some travel will be required over the course of the 2-year study. Further monitoring of participants will occur every year with tests that would be the standard of care for patients with retinal diseases.

**Possible Benefits:**

The information that is gained from the study will help us to understand if gene therapy for your child’s disease is safe. It will also help us to develop good methods for testing if gene therapy is effective in slowing or stopping vision loss. Your child’s participation will help us in our research, which aims to slow or possibly prevent further loss of sight that occurs in children affected by the same disease.

**Possible Risks:**

Gene therapy for genetic eye disease is new and there is not a lot of information about its risks in humans. To date no serious adverse events have occurred in the few patients enrolled in a similar clinical trials at universities in the United States and United Kingdom. However, possible risks are:

***Surgical Risks:*** All surgical procedures carry a risk of side effects. The possible surgical risks include tears or detachment of the retina, bleeding and infection. These complications can normally be treated effectively by medications or further surgery, but can sometimes result in a permanent loss of eyesight.

***Gene Therapy Risks:*** There is a remote possibility of an untreatable, serious effect that might result in complete loss of vision or loss of an eye. The modified virus carrying the new gene is developed from a virus (known as adeno- associated virus, AAV) that causes no known disease in humans. The virus has been disabled so that it cannot cause infection.

There is a small possibility that the modified virus may cause some inflammation inside the eye but this is considered unlikely based on evidence from adult patients treated in similar gene transfer trials. However, children are not little adults. We have no prior safety evidence for this gene in this procedure for child patients. We do, however, have safety evidence in children from the LCA gene therapy clinical trials, which started in 2013. We intend to minimize any risk of inflammation by asking your child to take steroid tablets for 3 days prior to the operation and for 1 week afterwards. Steroids can cause a range of effects that include a rise in blood sugar and blood pressure. We will be monitoring your child for these signs during this period. As well, if an inflammation does occur in the eye, it is unlikely to spread to the body and can be managed with steroid containing eye drops.

There is the small possibility that the modified virus carrying the working genes will insert itself at a location where it disrupts another gene, resulting in an increased risk of tumor formation. However, this result has not been observed in other trials using the same type of modified virus as in this trial. It is highly unlikely to occur in the cells of the eye. Follow-up testing is expected to identify this remote possibility at an early stage to enable quick and effective treatment.

Tiny amounts of the injected modified virus may spread along the optic nerve towards the brain. The surgical method has been designed to limit the effects of the gene therapy to the retina. Any risk of toxicity in the brain is very small. Researchers in another study injected a similar modified virus directly into the brain of 12 patients with a neurological disease without side effects.

In theory, gene therapy could affect the next generation. We consider the risk of the new gene being passed on to future children minimal since only tiny amounts of modified virus will be delivered within the eye. Gene therapy to the eye will not prevent your children’s children from being carriers of the disease; your grandchildren and future generations could still be affected by the same ocular disease as your child.

**Confidentiality:**

Personal records relating to this study will be kept confidential unless release is required by law. Absolute confidentiality cannot be guaranteed. Research data collected about your child for this study will not identify your child by name, only by initials and a coded number. Your child’s name will not be released outside the research clinic. Any report that we publish as a result of this study will not identify your child by name.

For this study, the doctor may need to access your child’s personal health records for health information, such as past medical history and test results. The doctor may also need to contact your family doctor and other health care providers to get additional information.

Health information collected for this study will be kept confidential and will be used only for the purpose of this research study. With your permission, your child’s study doctor will inform your family physician of your child’s participation in this study and your family doctor will be consulted regarding your child’s health and treatment.

The University of Alberta Health Research Ethics Board, Health Canada, and other regulatory agencies may have access to your child’s records to monitor the research.

By signing this consent form, you give permission for the collection, use, and disclosure of your child’s medical records. In Canada, study information is required to be kept for 25 years. Even if you withdraw from the study, the medical information obtained from your child for study purposes will not be destroyed. You have a right to check your child’s health records and request changes if their personal information is incorrect.

If you or your child withdraws consent from study for any reason, the study doctor may continue to use and disclose your child’s Personal Health Information collected prior to the time your child stopped taking part.

**Voluntary Participation:**

Participation in the study is entirely voluntary. You or your child can refuse to participate in the study, or you or your child can withdraw at any time from the study without needing to give a reason. Should either of you decide not to participate in this study, your and your child’s clinical care will be affected. If you or your child decide to withdraw at any time, data already collected may still be used for this study.

The only reason study staff would ask your child to withdraw from the study would be if continued participation would negatively affect their health.

Sometimes during the course of a research project, new information becomes available about the treatment/drug that is being studied. If this happens, the research doctor will tell you and your child about it and discuss whether the child should continue in the study.

**Costs and Compensation:**

Our research team will cover the costs of medications required during the course of this study and costs for parking and/or local transportation for surgical and follow-up visits.

**Compensation for Injury:**

If your child becomes ill or injured as a result of being in this study, they will receive necessary medical treatment, at no additional cost to you. By signing this consent form, you are not releasing the investigator(s), institution(s) and/or sponsor(s) from their legal and professional responsibilities.

----------------------------------------------------------------------------------------------------------------------------

**Information Sheet #2: An open label pediatric clinical trial of stem cell transplantation for type 1 diabetes mellitus**

**Background:**

Right now, there are no effective treatments available to restore blood glucose control without close self-monitoring by those living with type 1 diabetes. We are testing a new technique using insulin-producing cells to help re-introduce functioning pancreatic cells into the body of children affected by type 1 diabetes. We think this new technique may help control or eliminate the need to self-monitor blood glucose altogether. The new technique uses pancreatic cells (created from human embryonic stem cells) in a soft protective capsule, similar to a balloon, to protect the cells from the immune system to act as a new pancreas. This is done by surgically inserting the capsule with the cells inside it, underneath the skin. If successful, the cells will begin to produce insulin and release it through the capsule for your child without any outside monitoring or insulin delivery required. However, it may take several months before the capsule works on its own so you will continue to follow your current insulin regimen. Your child’s study doctor will monitor insulin needs and adjust prescriptions over the course of the study.

This method of stem cell transplantation has helped a small number of adult patients with type 1 diabetes but has never been tested in children. Clinical research with a small number of participants has shown that inserting the capsule and pancreatic cells under the skin of adult patients with type 1 diabetes can help regulate or restore the body’s ability to produce insulin without interference from the immune system. However, we do not know how this stem cell transplantation will affect child research participants. A stem cell transplantation trial, similar to this one began at a university in the United States three years ago. So far, nine participants with type 1 diabetes have undergone the capsule implantation surgery using the same method that we will use on your child.

**Purpose:**

The main purpose of this study is to make sure that the capsule and stem cell doses can be surgically inserted under the skin to treat pediatric type 1 diabetes and maintained safely for two years. The secondary purpose is to develop good methods for testing if the method is effective in treating type 1 diabetes in child populations. These will then be used in future stem cell transplantation clinical trials.

We will follow research participants 2 years total to find out if the stem cell transplantation is safe and useful for reducing insulin dependence. For this study, we intend to include 12 participants.

**Criteria for Participation:**

*Normally, this section would have more detail but for the purpose of this study, we will assume that your child fits the inclusion criteria to join if you chose to enroll him/her*.

**Procedure:**

As part of this stem cell transplantation study, your child will have an operation to implant the capsule carrying the pancreatic cells under their skin on their outer thigh. This procedure involves surgery using local anesthesia to the skin. The surgeon will insert the capsule under the skin by making an incision (cut) on your child’s skin. The capsule is similar in shape and size to a business card. The surgical procedure will be videotaped to provide a record of the operation and determine its safety.

Your child will also undergo a surgery to remove the implant after the study is complete. The surgery will be same for the implantation and removal in terms of anesthetic and size of the incision made.

Once your child is part of the study, he or she will have a series of initial tests (Table 1), most of which would have been performed as part of their routine diabetes care. The monitoring of the insulin production and immune system reactions involves standardized technical equipment for photography, blood glucose (laboratory blood samples), and insulin response measurements (intravenous catheter, i.e. and IV). If your family does not live in Alberta, as many tests as possible will be performed at your referring physician’s centre so that you do not have to travel as much. However some travel will be required over the course of the 2-year study.

**Possible Benefits:**

The information that is gained from the study will help us to understand if stem cell transplantation using this method for pediatric type 1 diabetes is safe. Information obtained from your participation may provide valuable information to assist future children with type 1 diabetes. During the study, your child’s diabetes will be closely monitored and they will receive medical care. However the child may not get any benefit from the capsule or the pancreatic cells that we are testing.

**Possible Risks:**

Pancreatic cell transplantation using a capsule like this is new and there is not a lot of information about its risks in children. To date, there have been no serious adverse events that have occurred in the few adult patients enrolled in a similar trial being undertaken through a university in the United States. However, child and adult bodies are not the same physically. Children are not little adults and unforeseen risks may arise from the surgery and from the implanted capsule.

**Surgical Risks:**

Implantation and removal of the capsule may cause your child pain, bleeding, seroma (a pocket of bodily fluid), tenderness, redness, and infection where the incisions were made. Also, if implanted incorrectly, the capsule could push against the skin causing pain. Surgeons are trained in the procedure and all necessary steps will be taken to minimize the risks and to make sure your child does not have a lot of pain during and after the surgery.

Local anesthesia (numbing) medications can sting or burn for a few seconds before numbing the skin. Other less likely side effects include nausea, vomiting, dizziness, drowsiness, local allergic reactions (redness, itching, and rash), low blood pressure, weakness, severe numbness or tingling, ringing in ears, blurry or double vision, slurred speech, metallic taste in mouth, mental status change, muscle twitching, and seizures.

**Monitoring Risks:**

If the capsule and cells begin working like a pancreas and produce insulin, it will be extremely important to monitor your child’s blood sugars closely with the study doctor and to lower insulin dosage when needed to avoid *low blood sugar*.

In case of low blood sugar, your child may feel the following symptoms: hunger, headache, dizziness, light-headedness, sweating, irregular heartbeat, racing heartbeat, shakiness, irritability, blurred vision, and disorientation not explained by another cause (for example, increased physical activity, skipped meal, etc.). In severe cases, loss‑of‑consciousness or fainting can happen where your child would need the help of someone else to make them feel better. You or your child must notify the study doctor right away if this happens.

We take blood to test insulin and other vital measurements throughout the study. Sometimes we will need more than one tube of blood during one visit. Your child may have bruising, irritation, pain, or redness where blood is drawn. An infection at the blood draw site is possible, but rare.

**Stem Cell Transplantation Risks:**

The purpose of this trial is to find out if the capsule and the transplanted pancreatic cells will work at all in a child population. We do not know whether the transplanted cells will ever function, let alone begin functioning quickly, to start working as a new pancreas for your child. Furthermore, we do not know how a child body will affect the durability of the capsule or how it will affect the capsule’s ability to protect the pancreatic cells from immune response. There is a risk that the capsule itself will not last two years. There is also a risk the implant may rupture.

There is a potential risk of immune and inflammatory reactions (how the body recognizes and defends itself from foreign materials) to the foreign capsule, though the capsule is designed to minimize these reactions. Some symptoms may include: rash or itching; shortness of breath, difficulty breathing, or wheezing; sudden drop in blood pressure, dizziness, or loss of consciousness; swelling around the skin, mouth, tongue, throat, or eyes; fast pulse or sweating; or new sudden pain in multiple joints. These could include anaphylactic reaction, which may be life-threatening if not treated immediately. If you think your child may be having an allergic reaction, you should immediately seek medical help. Also seek treatment immediately and tell your study doctor and study staff if your child has these or other symptoms during the study.

This type of research is invasive, very new, and experimental. Because scientists want to reduce anything that can confuse the findings of their studies, there is a risk that joining this research will disqualify your child from future islet cell or other tissue or organ transplantation research. Furthermore, because we do not know how the long-term interaction between this procedure and other standards of care, participation may disqualify your child from approved islet cell or other organ transplantation operations in general.

There is a risk of abnormal cell growth due to the stem cell technology (e.g., benign or cancerous tumors development). We have not seen this happen in the adult participants of prior studies, but again, children are not little adults and we do not know how they will react to this procedure. Ultrasound monitoring will be done during the study to watch for abnormal cell growth. If your study doctor believes there could be a growth, the units may be removed from your child’s body.

There is a risk for pregnant women to develop alloimmune responses (where the immune system attacks human cells that are introduced to the body. This reaction is particularly dangerous as the alloimmunization can cause severe complications in pregnancy that may result in the death of the fetus. We do not know how stem cell transplantation in males can impact pregnancy outcomes. Thus, we ask both males and females of childbearing potential to use two forms of birth control.

**Confidentiality:**

Personal records relating to this study will be kept confidential unless release is required by law. Absolute confidentiality cannot be guaranteed. Research data collected about your child for this study will not identify your child by name, only by initials and a coded number. Your child’s name will not be released outside the research clinic. Any report that we publish as a result of this study will not identify your child by name.

For this study, the doctor may need to access your child’s personal health records for health information, such as past medical history and test results. The doctor may also need to contact your family doctor and other health care providers to get additional information.

Health information collected for this study will be kept confidential and will be used only for the purpose of this research study. By signing the consent form you give permission to the study staff to access any personally identifiable health information held by other health care professionals, which is necessary for the conduct of the research. With your permission, your child’s study doctor will inform your family physician of your child’s participation in this study and your family doctor will be consulted regarding your child’s health and treatment.

The University of Alberta Health Research Ethics Board, Health Canada, and other regulatory agencies may have access to your child’s records to monitor the research and verify the accuracy of study data.

By signing this consent form, you give permission for the collection, use, and disclosure of your child’s medical records. In Canada, study information is required to be kept for 25 years. Even if you or your child withdraws from the study, the medical information obtained from your child for study purposes will not be destroyed. You have a right to check your child’s health records and request changes if their personal information is incorrect.

If you or your child withdraws consent from study for any reason, the study doctor may continue to use and disclose your child’s Personal Health Information collected prior to the time your child stopped taking part.

**Voluntary Participation:**

Participation in the study is entirely voluntary. You or your child can refuse to participate in the study, or you or your child can withdraw at any time from the study without needing to give a reason. Should either of you decide not to participate in this study, clinical care, for neither you nor your child, will be affected. If you or your child decides to withdraw at any time, data already collected may still be used for this study. The only reason study staff would ask your child to withdraw from the study would be if continued participation would negatively affect their health.

Sometimes during the course of a research project, new information becomes available about the treatment/drug that is being studied. If this happens, the research doctor will tell you and your child about it and discuss whether the child should continue in the study. If you or your child decide not to carry on, the research doctor will arrange for your child’s care to continue. If you and your child decide to continue in the study, you will be asked to sign an updated consent form and your child will be asked to sign an updated assent form.

**Costs and Compensation:**

Our research team will cover the costs of medications required during the course of this study and costs for parking and/or local transportation for surgical and follow-up visits.

**Compensation for Injury:**

If your child becomes ill or injured as a result of being in this study, they will receive necessary medical treatment, at no additional cost to you. By signing this consent form, you are not releasing the investigator(s), institution(s) and/or sponsor(s) from their legal and professional responsibilities.

**Information Sheet #3: Autologous Hematopoietic Stem Cell Mobilization, Transplantation and Immunologic Reset in New Onset Type 1 Diabetes Mellitus.**

**What is the reason for doing the study?**

Most Type 1 Diabetes (T1D) patients require insulin injections for the rest of their lives. We are studying one approach that may eliminate the need for insulin injections. This approach uses an autologous hematopoietic (blood) stem cell transplant combined with 5 medications. This approach has been tested in small number of adult patients with type 1 diabetes, but it **has never been tested in children.**

**What is this study testing?**

Autologous hematopoietic stem cell transplantation involves collecting a patient’s blood, isolating the stem cells, multiplying those stem cells in a laboratory, and then re-infusing the stem cells back into the same patient. Stem cells are very young cells that are found in the bone marrow. Blood stem cells have the potential to make all the different types of cells found in the blood system. To collect the stem cells, we first inject the patient with Medication A. Medication A moves the stem cells out of the bone marrow into the bloodstream (mobilizes the stem cells), so we can collect them from the bloodstream.

During blood stem cell collection, your child will be connected to an apheresis machine, also known as a cell separator. Blood will leave their body through a catheter placed in a large vein (known as a central venous catheter). The machine will separate out the stem cells and return the remaining blood components through the catheter. A blood thinner called citrate may be slowly added to your child’s blood during this process to help prevent blood clotting.

After the collection, your child’s stem cells will be cultured and stored till the time of the stem cell transplant. On the day of your child’s transplant, the previously collected stem cells will be infused into your child through his/her splenic artery so that they can take up residence in the pancreas (a process called homing). Infusion time depends upon the volume of cells to be infused.

The aim of this study is to test whether the infused stem cells grow and turn into (differentiate) insulin producing cells, which may eliminate your child’s need for daily insulin injections. Even though the stem cells start the homing process right away, it may take 6 months or more before these infused cells are able to mature and produce insulin.

**What will I be asked to do?**

If you and your child consent to take part in the study, your child will receive ***5 Health Canada approved medications - plerixafor along with alemtuzumab, etanercept, anakinra and liraglutide.*** These medications are believed to help the stem cells to mature and produce insulin faster.

During the study, your child will be asked to give routine blood samples on ten occasions over 2 years to test and characterize your child’s blood (for autoreactivity and T-cell phenotyping). The study will take about 24 months from the screening visit until the end of the study. You will be asked to attend about 12 scheduled Study Visits in addition to the screening visit (Participants will be provided with a detailed schedule for visits and procedures).

**What are the risks and discomforts?**

***Blood Collection:*** The risks of having blood taken include discomfort, bleeding, or bruising where the needle enters the skin.

***Stem Cell Collection:*** The most common bad reactions to stem cell collection have been: diarrhea, nausea, tiredness (fatigue), injection site reactions, headache, joint pain (arthralgia), dizziness, and vomiting.

***Mild and severe risks associated with the drugs:*** These range from mild-medium discomfort (e.g., vomiting, diarrhea) to life-threatening reactions and death. The consent document will provide a detailed account of these side-effects classified as mild and severe. We have included these in the attached appendix if you are interested in the types of risks.

**What are the benefits of the study?**

It is not known whether the experimental procedure will work or how long the transplant will eliminate the need to take insulin. The information learned from this study may improve the future treatment of others with T1D. However, your child may not get any benefit from being in this research study.

**Confidentiality:**

Personal records relating to this study will be kept confidential unless release is required by law. Absolute confidentiality cannot be guaranteed. Research data collected about your child for this study will not identify your child by name, only by initials and a coded number. Your child’s name will not be released outside the research clinic. Any report that we publish as a result of this study will not identify your child by name.

For this study, the doctor may need to access your child’s personal health records for health information, such as past medical history and test results. The doctor will also need to contact your family doctor and other health care providers to ensure proper care given the experimental treatments and medications your child will be taking.

Health information collected for this study will be kept confidential and will be used only for the purpose of this research study. By signing the consent form you give permission to the study staff to access any personally identifiable health information held by other health care professionals, which is necessary for the conduct of the research. With your permission, your child’s study doctor will inform your family physician of your child’s participation in this study and your family doctor will be consulted regarding your child’s health and treatment.

The University of Alberta Health Research Ethics Board, Health Canada, and other regulatory agencies may have access to your child’s records to monitor the research and verify the accuracy of study data.

By signing this consent form, you give permission for the collection, use, and disclosure of your child’s medical records. In Canada, study information is required to be kept for 25 years. Even if you or your child withdraws from the study, the medical information obtained from your child for study purposes will not be destroyed. You have a right to check your child’s health records and request changes if their personal information is incorrect.

If you or your child withdraws consent from study for any reason, the study doctor may continue to use and disclose your child’s Personal Health Information collected prior to the time your child stopped taking part.

**Voluntary Participation:**

Participation in the study is entirely voluntary. You or your child can refuse to participate in the study, or you or your child can withdraw at any time from the study without needing to give a reason. Should either of you decide not to participate in this study, clinical care, for neither you nor your child, will be affected. If you or your child decides to withdraw at any time, data already collected may still be used for this study. The only reason study staff would ask your child to withdraw from the study would be if continued participation would negatively affect their health.

Sometimes during the course of a research project, new information becomes available about the treatment/drug that is being studied. If this happens, the research doctor will tell you and your child about it and discuss whether the child should continue in the study. If you or your child decide not to carry on, the research doctor will arrange for your child’s care to continue. If you and your child decide to continue in the study, you will be asked to sign an updated consent form and your child will be asked to sign an updated assent form.

**Costs and Compensation:**

Our research team will cover the costs of medications required during the course of this study and costs for parking and/or local transportation for surgical and follow-up visits. If your child becomes ill or injured as a result of being in this study, they will receive necessary medical treatment, at no additional cost to you. By signing this consent form, you are not releasing the investigator(s), institution(s) and/or sponsor(s) from their legal and professional responsibilities.

**Appendix: Summary of Drug Risks (this list is not comprehensive of the risks):**

***Plerixafor:***Severe, life-threatening allergic reactions (anaphylaxis) can happen in people who take plerixafor.

- Thrombocytopenia (a decrease in the number of platelets circulating in the blood) has been observed in patients receiving plerixafor. Your platelet counts will be monitored.
- The spleen may be examined if your child experiences pain in the left upper stomach area or left shoulder area as these may be signs of an enlarged or burst (ruptured) spleen.

***Other Drugs:***

Alemtuzumab, etanercept and anakinra, are immunosuppresion drugs that may increase your chances of developing serious infections and certain cancers. Some of these infections or cancers can become life-threatening and if treatment fails, may lead to death. The combination of immunosuppressants used in this trial is experimental, but is in accordance with widely recommended current clinical standards.

Immunosuppression depletes cells that cause cell rejections but these cells also normally protect people from fungal or viral infections. Therefore, transplant patients are at increased risk for certain virus infections and fungal infections during immunosupression (usually between 2-6 months). All transplant patients receive other medication to prevent viral and fungal infection. These drugs are generally effective in preventing infection, but are not completely effective in all cases.

Because your child will be at risk of infection, they will not be allowed to receive ANY vaccinations for 2 months prior to cell transplantation and one year afterwards. This includes routine vaccinations such as tetanus, influenza (flu) or measles. This is because vaccinations work when the body makes antibodies to whatever is in the vaccine. Because your child will not be vaccinated, they may be at risk of developing these infections.

*Common side effects:*

- Low blood pressure
- Shortness of breath
- “Serum sickness” (a reaction with skin rashes, swelling of tissue, swollen glands, joint pain, fever, and lack of energy) is also possible.
- Many medicinal side effects are reduced substantially by the use acetaminophen and allergy medicine. You will therefore be given Benadryl and Tylenol prior to receiving your first dose of Medication B. Alemtuzumab may be associated with an increased risk of a type of autoimmune thyroid disease.

*Less common and rare side effects:*

- Severe, life-threatening allergic reactions (anaphylaxis)
- Thrombocytopenia (a decrease in the number of platelets circulating in the blood) has been observed in patients receiving one of the medications. Your child’s platelet counts will be monitored.
- Enlarged or burst (ruptured) spleen.
- Pain, decreased appetite, sweating, pneumonia, infection, muscle aches, weakness, high blood pressure, sore throat, abdominal pain, back pain, dizziness and anemia.
- Muscle tremors, cancer, immune-system damage to multiple organs, failure to produce red blood cells (which can be fatal), blurred vision, loss of bladder and/or bowel control and partial or complete paralysis (these neurological problems usually go away partially or completely after stopping drug use), in very rare cases serious infection and death have occurred.
- One medication may cause certain white blood cells called neutrophils to decrease in number (neutropenia), increasing risks of serious infections.
- Subjects on long-term administration of the medications may be at higher risk to develop malignancies (Lymphoma, a type of cancer).

***Risk for Liraglutide (blood glucose regulating medication):***

Liraglutide is a drug developed to help induce insulin production and control blood glucose in Type 2 Diabetes patients. Although Liraglutide has been given to thousands of type 2 diabetes patients, and some adults with T1D, it has not been used in conjunction with stem cell transplant in children.

*Possible side effects:*

- nausea, diarrhea, vomiting and constipation, and abdominal pain. These side effects are usually mild, usually last a short time and do not lead to discontinuation of treatment; they may go away after your child is on liraglutide for a few weeks and also when the drug is stopped.
- *Dehydration and decrease in kidney function:* Dehydration, caused by vomiting or diarrhea, may cause kidney failure which can lead to the need for dialysis. This can happen in people who have never had kidney problems before. Drinking plenty of fluids may reduce the chance of dehydration.
- *Hypoglycemia episodes (too low blood glucose)*
- *Hyperglycemia (too high blood glucose)*
- *Pancreatitis:* persistent severe abdominal pain (usually accompanied with vomiting) reported from long-term clinical trials.
- *C-cell tumor:* When liraglutide was given to rats and mice for most of their lifetime, it caused tumors, called "C-cell tumors", of the thyroid gland. Some of these tumors were cancers. It is not known whether liraglutide will cause C-cell tumors or cancer in people.
